# Assessing reader reception of Arthur Waley’s English translation of *Journey to the West*: A topic modeling and sentiment analysis approach

**DOI:** 10.1371/journal.pone.0351327

**Published:** 2026-06-26

**Authors:** Jingting Zeng, Tianchi Qiu, Linmin Shan

**Affiliations:** School of Foreign Languages, Jiangsu University of Science and Technology, Zhenjiang, China; University of Kerbala, IRAQ

## Abstract

Arthur Waley’s 1942 abridged translation, *Monkey: Journey to the West*, remains one of the most influential and widely debated English renderings of this Chinese classic. Despite its extensive global cultural impact, the contemporary online reception has remained largely unexplored. This study applies an integrated computational framework — combining BERTopic topic modelling, Twitter-RoBERTa sentiment classification, and GPT-4o-assisted qualitative validation — to analyse 796 English-language reader reviews of Waley’s translation collected from Amazon and Goodreads. The study aims to systematically investigate the contemporary online reception, identify the thematic concerns and interpretive patterns of English-language readers, exploring the dynamics of its cross-cultural dissemination. The findings indicate that *Monkey* is generally well received among international readers (43.12% positive sentiment), driven by appreciation for Waley’s colloquial prose, Sun Wukong’s appeal as a trickster-hero, and the translation’s cross-cultural philosophical resonance and humour. Conversely, negative feedback (18.30%) is associated with structural tensions with Western narrative and heroic conventions, insufficient paratextual guidance for cultural interpretation, and ineffective multimodal delivery — most notably, British-accented audiobook narrations. The study concludes that the global circulation of *Monkey* is shaped by a complex interplay of translation strategies, reader cultural schemas, and multimodal delivery formats. Furthermore, this hybrid methodological approach offers a replicable empirical framework for investigating international reception of Chinese literary classics in the digital era.

## 1. Introduction

The reception of translated classics illuminates cross-cultural dialogue and global narrative circulation [1,2]. *Journey to the West*, one of the “Four Great Classical Novels” of China, combining myth, fantasy, and socio-religious allegory, is viewed as a paradigmatic case, in particular through Arthur Waley’s highly influential 1942 abridgment, *Monkey*. Waley’s acclaimed *Monkey*—praised by Edith Sitwell and *The Times*—achieved extensive cultural impact, yet has also generated sustained debate over translation ethics and textual fidelity, owing to its substantial abridgment and cultural reframing.

Traditionally, translation studies have prioritized source-text influence and textual strategies, leaving reader reception comparatively marginalized. Following the cultural turn in translation studies, scholarly interest has shifted toward the active role of readers in the process of meaning-making [3]. The way diverse contemporary audiences engage with an influential yet contested text like Monkey fundamentally shapes its cross-cultural legacy; however, this dimension remains significantly underexplored. Existing research has extensively scrutinized Waley’s domesticating strategies [4], his colloquial prose style [5], the work’s overall readability [6], the social networks facilitating its canonization [7], and the cultural nuances lost through abridgment [8]. Yet, these studies remain largely production-oriented, anchored in the analysis of the text itself. Consequently, a systematic, empirical account of how ordinary readers actually perceive and respond to the translation is still lacking.

This methodological gap stems from the inherent complexities of reader reception research, which is interdisciplinary, subjective, and characterized by vast data volumes. Digital humanities (DH) methodologies offer a robust pathway forward. This study constructs a computational framework combining topic modeling and sentiment analysis to conduct a large-scale empirical investigation into the contemporary online reception of *Monkey*. By leveraging BERTopic to map thematic landscapes and structural associations within reader reviews, and deploying the Twitter-RoBERTa model to quantify sentiment polarities and distributional variances, we provide a multi-dimensional analysis of reader engagement. Accordingly, this study addresses the following research questions (RQs):

Question 1: What are the primary thematic landscapes that characterize the online reception of Waley’s *Monkey*, and what underlying structural relationships exist among these themes?

Question 2: How are sentiment polarities distributed across these topics, and what specific distributional variances can be observed?

Question 3: What factors drive the divergence in reader sentiment, and how does the interplay between topics and sentiment reveal the dynamics of the translation’s cross-cultural reception?

## 2. Literature review

### 2.1. Relevant studies on reception of the English translations of *Journey to the West*

Existing scholarship on English translations of *Journey to the West* — focusing primarily on versions by Arthur Waley (1942), Anthony C. Yu (2012), and Julia Lovell (2021) — has developed along three main areas: the analysis of internal textual features [4,6], the scrutiny of institutional networks [7,9], and burgeoning empirical research grounded in reader feedback [10].

Initial research focused on how textual construction mediates the reading experience. These studies established that cross-cultural mediation strategies [4] and systematic narrative abridgment [6] reduce cognitive load for target readers by aligning the text with their cultural expectations. More recently, scholars employing computational stylistics and narrative reframing [8,11] have further quantified translational style, elucidating the textual mechanisms that govern contemporary audience perception.

Beyond the text itself, scholars have investigated the institutional forces that preconfigure reception. Drawing on Actor-Network Theory and archival evidence, Luo and Zheng [7,9] reconstructed the historical conditions driving the work’s canonization, highlighting how publishing mechanisms intervene in textual production. Similarly, Qi’s archival analysis [12] documented the role of editorial gatekeeping in commercial negotiations. Collectively, these studies underscore the extent to which a translation’s reception is contingent upon institutional power and mediation.

Most recently, reception studies have taken an empirical turn. At the macro level, Wang et al. [13] used bibliometric analysis to demonstrate that existing scholarship disproportionately reflects elite academic reception, thereby marginalizing ordinary readers. At the micro level, Wang and Humblé [10] applied Qualitative Data Analysis (QDA) to online reviews, facilitating closer engagement with actual audience responses.

Taken together, however, these trajectories reveal a persistent methodological impasse. While the field has recognized the imperative of integrating real reader voices, analytical methodologies have not kept pace. Traditional approaches remain tethered to scholarly interpretation and idealized reader models, while existing empirical work — constrained by the limitations of manual coding and researcher subjectivity — struggles to process the vast volume of digital feedback. This study addresses this gap by introducing a computational framework from Digital Humanities, leveraging topic modeling to systematically mine large-scale reader reviews and provide a robust empirical foundation for understanding the global reception of *Journey to the West*.

### 2.2. Relevant studies on sentiment analysis in reader reception

Sentiment Analysis (SA), also known as opinion mining, is a widely used computational method in Natural Language Processing (NLP) that identifies and quantifies affective states and subjective stances within textual data [14]. Originally applied in commerce and information science [15,16], SA has since expanded into the humanities and social sciences [17], where its capacity to process large volumes of reader-generated text has opened new avenues for empirical reception research [18]. By bridging “distant reading” [19] with “close reading,” SA injects empirical vigour into domains like Translation Reception Studies (TRS). In TRS, SA has been applied to evaluate reader sentiment toward specific translations and to compare cross-cultural reception patterns. Previous studies in this area include: (1) identification of affective patterns in monolingual reviews of classics such as *Fortress Besieged* [20]; (2) application of deep learning models to assess translational impact in works like D.C. Lau’s *The Analects* [21]; and (3) use of cross-linguistic SA on texts such as *Wolf Totem* to examine how cultural schemata influence reception [22]. These approaches have collectively advanced TRS toward more quantitative and systematic analysis of reader engagement.

Methodologically, early SA relied on dictionary-based tools (e.g., syuzhet, VADER, TextBlob) and lexical-statistical techniques (e.g., LIWC). While accessible, these tools are limited in detecting contextual negations, implicit affect, and the nuanced semantics of culturally specific texts such as literary reviews [23–25]. To address these constraints, subsequent work has shifted toward Transformer-based machine learning models such as BERT, RoBERTa, and Large Language Models (LLMs) like GPT-4o, which offer superior contextual semantic representation [26].

However, applying these models to reception studies introduces a further challenge: standard architectures are pre-trained on formal corpora such as Wikipedia and published books, making them less suited to the informal, colloquial language of online reviews. Since *Monkey*’s reader reviews constitute user-generated content (UGC) characterized by internet slangs and fragmented syntax, this study addresses this domain mismatch by deploying Twitter-RoBERTa, a model pre-trained on large-scale social media data. Comparative fine-tuning experiments were conducted to optimize model parameters, enhancing sentiment quantification accuracy and bridging a key methodological gap in empirical translation reception research.

### 2.3. Relevant studies on topic modeling in reader reception

Topic Modeling (TM) is an unsupervised machine learning technique used in digital humanities to automatically extract underlying thematic structures from large textual datasets [27]. In literary reception studies, TM enables scholars to systematically identify prominent themes and engagement patterns within vast reader-generated content, such as online comments and discussion forums [28].

Its value in this domain is well established. Walsh et al. [29] applied Latent Dirichlet Allocation (LDA) to large-scale online reviews, revealing how the “classics industry” — encompassing school curricula and screen adaptations — shapes the literary cognition of general readers. Cui et al. [30] used WordStat to examine the overseas digital reception of *Wolf Totem*, finding that Western readers favored universal ecological themes — a result that validated the translator’s strategy of omitting didactic content and extended reception studies into the domain of large-scale “distant reading.”

Although classic TM methods like Latent Dirichlet Allocation (LDA) and Non-negative Matrix Factorization (NMF) being well-established, they are constrained by their reliance on surface-level word frequencies and a context-free “bag-of-words” assumption, limiting their ability to capture the nuanced semantics of complex literary texts [31]. To address this semantic limitation, combining TM with SA offers a complementary solution: while TM reveals which thematic aspects the readers discuss, SA quantifies how the readers feel about those aspects, providing a richer and more layered picture of reception.

Building on these advances, this study proposes a computational framework integrating BERTopic and sentiment analysis to examine the Anglophone digital reception of Arthur Waley’s *Monkey* (1942). BERTopic’s transformer-based embeddings overcome the semantic limitations of traditional frequency models by generating highly context-aware topics. The combined framework shifts reception analysis from one-dimensional thematic overviews to a two-dimensional paradigm coupling topic identification with sentiment quantification, offering a replicable approach for decoding the cross-cultural reception of translated literature in digital contexts.

## 3. Research design

**Fig 1:**
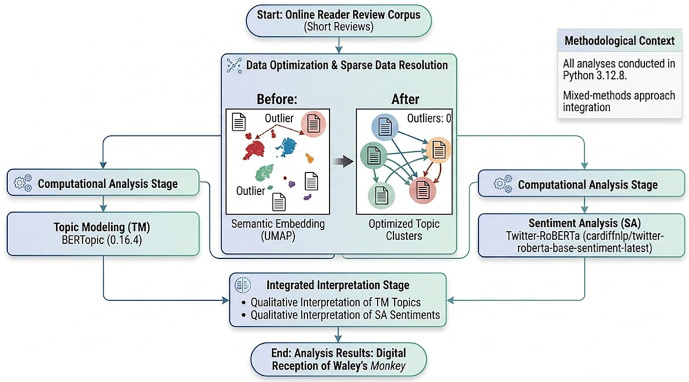
Workflow of research design.

This study undertakes a multi-faceted investigation into the digital reception of Waley’s seminal translation, *Monkey: Journey to the West*, through a rigorous analysis of a corpus online reader reviews. The research architecture, shown in Fig 1, adopts a mixed-methods approach, integrating advanced computational techniques—specifically, TM via BERTopic(0.16.4) and SA using Twitter-RoBERTa (cardiffnlp/twitter-roberta-base-sentiment-latest)—with detailed qualitative interpretation. A salient methodological contribution of this research is the explicit application of a Popularity Deviation Regularizer (PDR) and a Probabilistic Reassignment Matrix (PRM) to address data sparsity in short reviews, thereby enhancing the empirical foundation of the subsequent TM and SA. All analyses were conducted in Python 3.12.8.

### 3.1. Data collection and corpus construction

The empirical foundation of this study is a corpus of English-language reader reviews related to Waley’s *Monkey: Journey to the West*, systematically collected from two dominant digital platforms: Goodreads and Amazon. Functioning as “alternative ecosystems” for community engagement, these platforms empower users to share organic reading experiences and negotiate identities through public discourse [32]. Consequently, they serve as vital arenas for literary reception studies, providing extensive, publicly articulated meta-discursive critical commentary that offers valuable insights into collective hermeneutic practices and consumption patterns [33].

Review data were programmatically collected via the Web Scraper browser extension, specifically targeting entries corresponding to Waley’s translation. The temporal scope of data collection was restricted to reviews posted prior to January 15, 2026. This procedure yielded a preliminary corpus comprising 697 reviews from Goodreads and 99 reviews from Amazon. All collected information is publicly accessible and does not entail access to private user information, ensuring the research’s adherence to established ethical standards for using publicly available data.

### 3.2. Text preprocessing protocol

Following aggregation, the English-language review corpus underwent a systematic, multi-staged preprocessing protocol to optimize data quality and ensure analytical consistency. The process began with the identification and removal of duplicate reviews. Subsequently, textual normalization was performed using standardized procedures implemented with Python libraries, specifically the Natural Language Toolkit (NLTK 3.9.2). This normalization included: (1) converting all text to lowercase for uniformity; (2) expanding common English contractions (e.g., “can’t” to “cannot”) using a predefined dictionary of 24 mappings; and (3) removing Uniform Resource Locators (URLs), most special characters (while preserving essential apostrophes), and redundant whitespace via regular expressions. These comprehensive preprocessing steps resulted in a refined, standardized dataset, ready for the subsequent LLM-driven data augmentation, BERTopic-based TM, and Twitter-RoBERTa-based SA.

### 3.3. Innovative data augmentation: Addressing data sparsity in short reviews

A distinct characteristic of online review datasets is the prevalence of extremely brief texts. This structural brevity and semantic isolation cause severe data sparsity, preventing texts from meeting minimum density thresholds for effective clustering. In BERTopic, this manifests as a massive volume of unassigned texts categorized as “noise” or Topic −1 [34]. Such sparsity poses a critical challenge to computational analyses like TM and SA.

To overcome this, this study implemented a mathematically transparent algorithmic refinement. This optimization leverages BERTopic’s modular architecture, which “allows researchers to customize each step without affecting the others” [32]. Given the complex noise in current reception data—driven by both brevity and semantic dispersion—the standard outlier reduction function [35] proved insufficient, necessitating an upgraded multi-module strategy.

First, to mitigate theme homogenization caused by high-frequency generic terms, a Popularity Deviation Regularizer (PDR) was introduced. This applies an exponential decay based on term rank to suppress generic terms while highlighting domain-specific vocabulary [36]. The PDR weight w(t) is formulated as (Equation 1):


$$\begin{array}{c} w(t)=TF-IDF(t)\times e^{-\alpha \cdot rank(t)} \end{array}$$ (1)

This ensures the model prioritizes unique lexical signatures related to *Journey to the West* over functional fillers.

Second, to recover informative texts missed by density-based clustering, a Probabilistic Reassignment Matrix (PRM) was implemented to “reallocate outlier documents to their nearest topic clusters using cosine similarity” [36]. The conditional probability $P\left(t_{j}|d_{i}\right)$ for this soft reassignment derives from semantic distance (Equation 2):


$$\begin{array}{c} P\left(t_{j}|d_{i}\right)=\frac{𝐞𝐱𝐩\left(-d_{mreach}\left(d_{i},t_{j}\right)\right)}{\sum_{k=1}^{M}𝐞𝐱𝐩\left(-d_{mreach}\left(d_{i},t_{k}\right)\right)} \end{array}$$ (2)

Empirical evidence confirms this refinement “effectively reduces invalid outlier assignments and preserves the semantic integrity of the corpus” [36].

Finally, the Maximal Marginal Relevance (MMR) method was utilized during keyword extraction “to promote term diversity and avoid redundancy” [36]. Collectively, these structural optimizations substantively enriched the dataset’s signal-to-noise ratio. This, in turn, enhanced the qualitative integrity of the input for the subsequent TM and SA phases and bolstered the generalizability of the study’s findings.

### 3.4. Topic modeling using BERTopic

**Fig 2:**
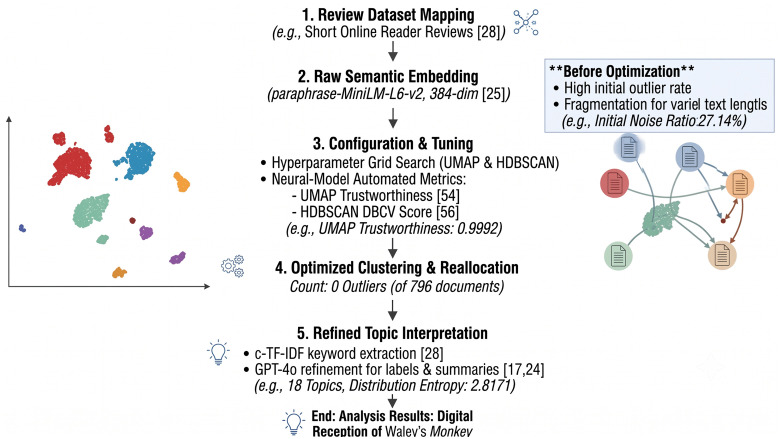
BERTopic Algorithm Components.

To shed light on the implicit thematic patterns of the reader review dataset, the current study implemented an optimized BERTopic pipeline [35], a sophisticated TM method that drew on transformer-based sentence embeddings to form semantically valid topic clusters. By doing this, it distinguishes itself from traditional methods like Latent Dirichlet Allocation (LDA) and Non-Negative Matrix Factorization (NMF). The detailed workflow (Fig 2) began by mapping reviews to dense vector representations utilizing the paraphrase-MiniLM-L6-v2 SBERT model. This specific model was selected for its superior balance between semantic richness and computational efficiency; as a distilled transformer-based model, it generates 384-dimensional vectors specifically optimized for paraphrase detection and semantic similarity tasks [34,37]. Given the fragmented semantic landscape of the reviews, the model’s ability to capture deep contextual meaning within a condensed vector space proved critical for processing heterogeneous text lengths.

Initially, the application of BERTopic with default parameters produced a high outlier rate that impacted topic clustering. A comprehensive grid search for hyperparameter tuning in terms of the UMAP and HDBSCAN algorithms to optimize configuration was conducted. This process utilized automated metrics specifically suited to BERTopic’s neural architecture: UMAP’s Trustworthiness [38] and HDBSCAN’s Density-Based Clustering Validation (DBCV) score [39]. These parameters were chosen as they are considered more reliable than traditional NPMI for neural models and more feasible than extensive human evaluation [31,35,40]. The iterative tuning identified an optimal configuration (UMAP: n_neighbors = 10, min_dist = 0.0, HDBSCAN:min_cluster_size = 15, min_samples = 2, min_topic_size = 20). This framework achieved an exceptional UMAP trustworthiness score of 0.9992, indicating that the low-dimensional mapping almost perfectly preserves the original semantic relationships.

Crucially, while the initial noise ratio during the parameter search was 27.14%, the subsequent integration of the PRM strategy effectively reallocated outliers based on embedding similarity. Consequently, the final model achieved a count of 0 outliers among the 796 documents. This total reallocation ensures that every reader’s voice is quantitatively accounted for in the thematic landscape. Ultimately, the optimized model yielded 18 well-defined topics with a distribution entropy of 2.8171, suggesting a highly balanced and diverse topic coverage.

**Fig 3:**
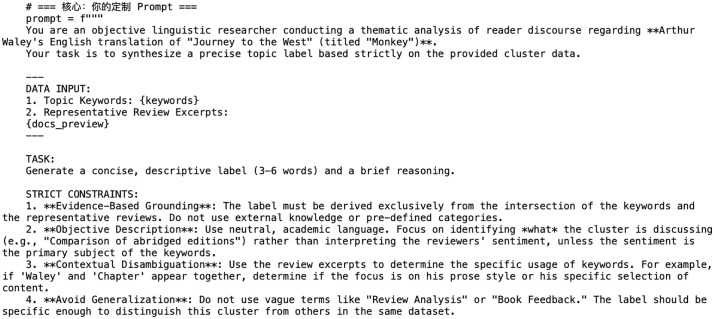
Label Condensing Prompt.

In the final analytical step, to ensure interpretive depth, GPT-4o was utilized to generate detailed topic labels and generate synthesized descriptions from the c-TF-IDF keywords [35] (Fig 3). This LLM-assisted refinement aligns with contemporary research advocating for LLMs to improve topic interpretability and provide context-rich summaries [41,42], thereby transforming keyword lists into more holistically understood thematic constructs.

### 3.5. Sentiment analysis using Twitter-RoBERTa

This study utilizes the Twitter-RoBERTa model for sentiment analysis. Pre-trained on approximately 124 million social media posts, the model exhibits exceptional domain adaptability for processing UGC found on platforms like Goodreads and Amazon. This extensive pre-training equips the model to effectively navigate the informal grammar, irony, and colloquial expressions prevalent in reader reviews.

To determine whether domain-specific fine-tuning would improve performance over native pre-training, a benchmarking comparison was conducted following [22]:

**Group 1 (Pre-trained):** The standard pipeline model was applied directly for inference without additional training, classifying sentences into three classes: negative, neutral, and positive.

**Group 2 (Fine-tuned):** The model was fine-tuned on the Stanford Sentiment Treebank (SST-5) dataset [43]. SST-5 provides fine-grained sentiment labels derived from continuous phrase-level scores. Labels were mapped to five sentiment classes as follows: very negative (*s* < 0.2), negative (0.2 ≤ *s* < 0.4), neutral (0.4 ≤ *s* < 0.6), positive (0.6 ≤ *s* < 0.8), and very positive (*s* ≥ 0.8). The official SST-5 train/validation/test split was observed (split-set labels 1/2/3 respectively). Fine-tuning hyperparameters are reported: base model = twitter-roberta-base, learning rate = 2 × 10 ⁻ ⁵, epochs = 3, batch size = 16, maximum sequence length = 128, weight decay = 0.01, model selection strategy = load_best_model_at_end (validation accuracy). The five predicted classes were subsequently merged into three (positive, neutral, negative) to reduce noise from blurred class boundaries.

To validate predictive performance, a robust gold-standard dataset was established: two trained researchers independently annotated a random sample of 100 sentences from the review corpus. This manual annotation yielded perfect inter-coder reliability (Cohen’s kappa = 1) [44]. The empirical results indicate that Group 1 significantly outperforms Group 2 across all key metrics, achieving an accuracy of 89% compared to 48% (Table 1). This paradigm demonstrates that the native pre-training of Twitter-RoBERTa on vast UGC datasets provides superior domain adaptability for capturing the “organic expressions” of readers in digital spaces. The degraded performance of the fine-tuned model suggests that the specific linguistic features of literary reviews align more closely with general social media discourse than with standardized review datasets like SST-5. Consequently, the Group 1 configuration was definitively adopted for the main analysis of the entire corpus to guarantee maximum accuracy.

**Table 1:** Performance comparison of sentiment analysis models.

Model	Accuracy	Precision	Recall	F1_Score	Kappa_Score
**Model_Pre-trained**	0.89	0.9195	0.89	0.893	1
**Model_Finetuned**	0.48	0.3829	0.48	0.4208	1

## 4. Results & discussion

This section presents and discusses English-language reader reception of Arthur Waley’s translation of *Journey to the West* through a structured,multi-layered approach. Section 4.1 elaborates on the topic landscape generated by BERTopic modeling, with topics subsequently refined and annotated using GPT-4o and explores the structural interrelationships among them. Section 4.2 analyses reader sentiment at two levels: a corpus-wide overview of the overall sentiment distribution, and a quadrant-based examination of how sentiment varies across distinct topic groupings. Section 4.3 then integrates these analytical layers into a multidimensional framework, mapping the intersection of topic distribution and sentiment patterns. Building on this framework, Section 4.4 draws on close reading of individual reviews to explore the qualitative mechanisms underlying the reception patterns identified.

### 4.1. Topic landscape and identification of core topics in English reader reviews

#### 4.1.1. Topic modeling and landscape overview.

BERTopic yielded 18 primary topics from the corpus of 796 English reader reviews of translated Journey to the West. Each topic is characterized by its ten most representative keywords, accompanied by interpretive labels and rationales derived from these keywords and associated review content (Table 2).

**Table 2:** Keywords, labels and reasoning across topic.

Topic	Frequency	Thematic Label	Name	Representation
**0**	93	Comparison of Abridged Translations	0_translation_waley_version_read	[‘translation’, ‘waley’, ‘version’, ‘read’, ‘story’, ‘chapter’, ‘abridged’, ‘much’, ‘yu’, ‘west’]
**1**	80	“Cultural Impact and Adaptation of ‘Monkey’”	1_monkey_story_heaven_one	[‘monkey’, ‘story’, ‘heaven’, ‘one’, ‘book’, ‘power’, ‘journey’, ‘india’, ‘tale’, ‘king’]
**2**	71	Abridged Version’s Energetic Adventure	2_monkey_read_book_story	[‘monkey’, ‘read’, ‘book’, ‘story’, ‘character’, ‘would’, ‘like’, ‘fun’, ‘version’, ‘adventure’]
**3**	37	“Cultural and Literary Context of ‘Journey to the West’”	3_journey_wukong_sun_west	[‘journey’, ‘wukong’, ‘sun’, ‘west’, ‘chinese’, ‘buddhist’, ‘china’, ‘story’, ‘buddhism’, ‘tang’]
**4**	49	Comparison of “Monkey” and TV Adaptations	4_story_tv_read_adventure	[‘story’, ‘tv’, ‘read’, ‘adventure’, ‘show’, ‘enjoyed’, ‘feel’, ‘series’, ‘version’, ‘could’]
**5**	33	Positive Reception of Humorous Elements	5_fun_ok_great_fascinating	[‘fun’, ‘ok’, ‘great’, ‘fascinating’, ‘good’, ‘delicious’, ‘sillier’, ‘goofier’, ‘robust’, ‘goooood’]
**6**	42	“Cultural Adaptation of Classic Tale”	6_monkey_chinese_book_tale	[‘monkey’, ‘chinese’, ‘book’, ‘tale’, ‘read’, ‘reading’, ‘time’, ‘classic’, ‘character’, ‘original’]
**7**	46	Abridged Translation and Cultural Context	7_chinese_translation_version_read	[‘chinese’, ‘translation’, ‘version’, ‘read’, ‘book’, ‘classic’, ‘would’, ‘story’, ‘reading’, ‘like’]
**8**	34	“Fairy Tale Elements in Epic Narrative”	8_time_story_tale_fairy	[‘time’, ‘story’, ‘tale’, ‘fairy’, ‘collection’, ‘got’, ‘great’, ‘baby’, ‘star’, ‘epic’]
**9**	31	Exploration of Chinese Cultural Classics	9_chinese_asian_culture_classic	[‘chinese’, ‘asian’, ‘culture’, ‘classic’, ‘literature’, ‘novel’, ‘china’, ‘great’, ‘folk’, ‘favourite’]
**10**	45	“Mythological and Satirical Elements in ‘Monkey’”	10_like_story_tale_chinese	[‘like’, ‘story’, ‘tale’, ‘chinese’, ‘monkey’, ‘fairy’, ‘religion’, ‘buddhist’, ‘folk’, ‘god’]
**11**	34	Positive Reception of Waley’s Translation	11_read_interesting_fun_amazing	[‘read’, ‘interesting’, ‘fun’, ‘amazing’, ‘best’, ‘reading’, ‘classic’, ‘great’, ‘hilarious’, ‘unabridged’]
**12**	42	Abridged Translation of “Journey to the West”	12_book_read_story_monkey	[‘book’, ‘read’, ‘story’, ‘monkey’, ‘one’, ‘journey’, ‘west’, ‘much’, ‘page’, ‘character’]
**13**	24	“Monkey’s Mischief and Resolution Dynamics”	13_monkey_dear_fun_little	[‘monkey’, ‘dear’, ‘fun’, ‘little’, ‘as’, ‘satisfying’, ‘resolution’, ‘beat’, ‘mischief’, ‘came’]
**14**	32	Influence of Buddhism in Waley’s Translation	14_mind_book_mean_budda	[‘mind’, ‘book’, ‘mean’, ‘budda’, ‘chinese’, ‘buddhism’, ‘buddhist’, ‘could’, ‘journey’, ‘also’]
**15**	29	“Cultural and Mythological Context of ‘Monkey’”	15_monkey_journey_chinese_story	[‘monkey’, ‘journey’, ‘chinese’, ‘story’, ‘one’, ‘book’, ‘china’, ‘sun’, ‘monk’, ‘wukong’]
**16**	33	Evaluation of Humor and Writing Style	16_book_ever_funny_written	[‘book’, ‘ever’, ‘funny’, ‘written’, ‘one’, ‘read’, ‘year’, ‘entertaining’, ‘greatest’, ‘interesting’]
**17**	41	Enjoyment of Reading “Monkey” by Waley	17_read_book_enjoyed_page	[‘read’, ‘book’, ‘enjoyed’, ‘page’, ‘fun’, ‘time’, ‘love’, ‘college’, ‘really’, ‘chapter’]

#### 4.1.2. Interrelationships among topics.

**Fig 4:**
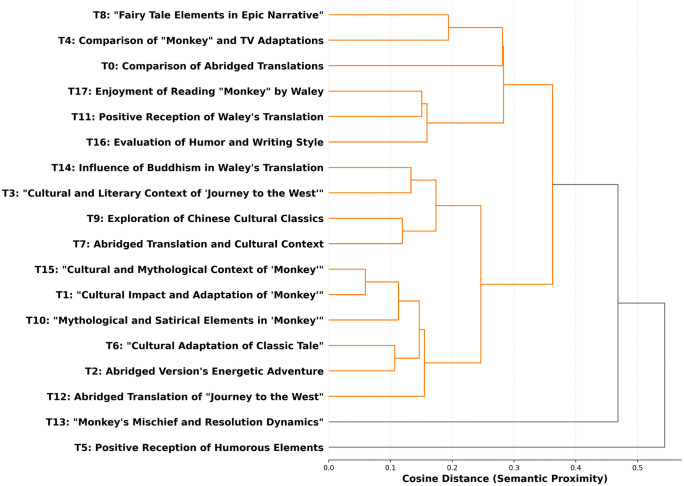
Dendrogram of Hierarchical Clustering.

**Fig 5:**
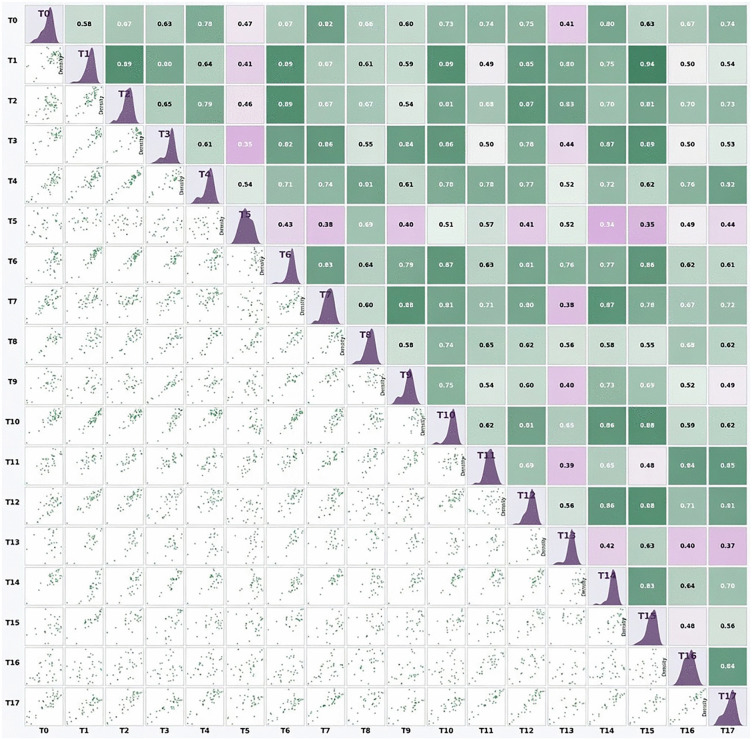
Cosine Similarity Matrix Across Topics.

To examine the structural interrelationships among these reader-perceived themes, hierarchical clustering analysis was applied to the topic embeddings. The resulting dendrogram (Fig 4) reveals clear semantic clusters and exposes a latent thematic structure of reader reception. This structure received further validation through a Cosine Similarity Matrix (Fig 5) mapping the topics based on semantic similarity.

Both analyses consistently identify strong thematic groupings. A prominent cluster integrates topics related to the reading experience and aesthetic appreciation of Waley’s prose style — specifically, enjoyment of the reading process and evaluation of his humorous English writing (Topics 11, 16, 17) — reflecting sustained reader engagement with the translator’s distinctive literary voice. A second significant cluster combines topics concerning Buddhism, Chinese cultural context, and broader historical background (Topics 3, 7, 9, 14), highlighting reader sensitivity to the relationship between the text’s religious dimensions and its cultural authenticity.

The largest cluster links readers’ interest in the work’s cultural impact, mythological elements, and the structural features of the abridged narrative (Topics 1, 2, 6, 10, 12, 15), indicating a consistent thematic intersection between the mythological journey and its translated presentation. A final cluster groups discussions comparing the text with television adaptations and its classification within the fairy tale genre (Topics 0, 4, 8), suggesting that readers draw on external media as cognitive reference points for interpreting the classic work.

The cosine similarity matrix (Fig 5) provides quantitative support for these groupings: the high similarity coefficient of 0.89 between Topics 1 and 6, for instance, confirms their shared position within the mythological domain. Topics 5 and 13 emerged as distinct outliers, with their isolation reflecting how the discrete character of experiential commentary relative to the broader thematic clusters. Taken together, these interrelationships indicate that reader reception is a structured system shaped by the interplay between the source text and the translator’s stylistic decisions.

#### 4.1.3. Topic stratification and quadrant-based analysis.

To evaluate the 18 identified topics systematically, this study assesses each along two dimensions: topic prevalence (review frequency) and topic distinctiveness (lexical significance). Topic distinctiveness is operationalized through a ‘Topic Keyword Intensity’ score (Table 3), calculated as the aggregated c-TF-IDF weights of a topic’s top N keywords, as identified by BERTopic [35]. A keyword’s c-TF-IDF score reflects its discriminative importance for a given topic relative to all others— a principle rooted in established Information Retrieval term-weighting methods [45]. While not a conventional criterion, such as topic coherence or diversity [46], this aggregate score provides a quantified measure of a topic’s collective lexical distinctiveness, analogous to TF-IDF-based document ranking: a higher Topic Keyword Intensity score indicate a topic characterized by a more discriminative keyword set (Equation 3):

**Table 3:** Intensity across topics.

Topic	Topic Intensity
5	1.1379384955003600
11	0.7870175770163970
16	0.6855451932670610
8	0.6562496198275580
9	0.614072589188527
13	0.5846489847713120
17	0.5047324026952140
7	0.3502145565681760
4	0.3393045773035720
6	0.3284082380625810
0	0.3233484356721500
2	0.2664224226421230
12	0.230219664443535
3	0.2145672421575780
14	0.21388264311759800
15	0.19181866340120
1	0.1915253760069440
10	0.1837904506558290


$$\begin{array}{c} Intensity_{t}=\sum_{kw\in K_{t}}w_{kw,t} \end{array}$$ (3)

The formula calculates the total intensity at time t. Here, t represents the time period, Kt represents the set of keywords active at time t, $w_{kw,t}\;$ represents the weight of keyword kw at time t, and $Intensity_{t}$ represents the aggregated intensity value.

### 4.2. Landscape, pattern and interpretation of sentiment in English reader reviews

Building on the topic identification in Section 4.1, this section conducts a transparent SA of reader reviews. We will first present the overall three-category sentiment distribution across the corpus, and subsequently use the quadrant framework to examine how sentiment varies across topic groupings and assess the statistical significance of observed differences. This multi-faceted approach aims to characterize the differentiated affective responses and the dynamic interplay between reader engagement and emotional responses.

#### 4.2.1. Initial corpus overview: Landscape of reader sentiment.

The sentiment profile constructed from 796 English reader reviews (4,207 analytical units) indicates a predominantly positive reception of Waley’s translation (Table 4). Positive responses account for 43.12% of the dataset, constituting a clear majority. Negative responses represent a substantially lower proportion (18.30%), while neutral responses account for 38.58%. This positive skew indicates a broadly appreciative engagement with the translated work among English-language readers. The comparatively modest share of negative commentary further supports this characterization of generally favourable reception.

**Table 4:** Overall sentiment distribution across the corpus.

Analysis Level	Sentiment Polarity	Count	Percentage (%)
Overall Corpus	Positive	1814	43.12%
Overall Corpus	Neutral	1623	38.58%
Overall Corpus	Negative	770	18.30%

#### 4.2.2. Quadrant-based landscape of reader sentiment.

**Fig 6:**
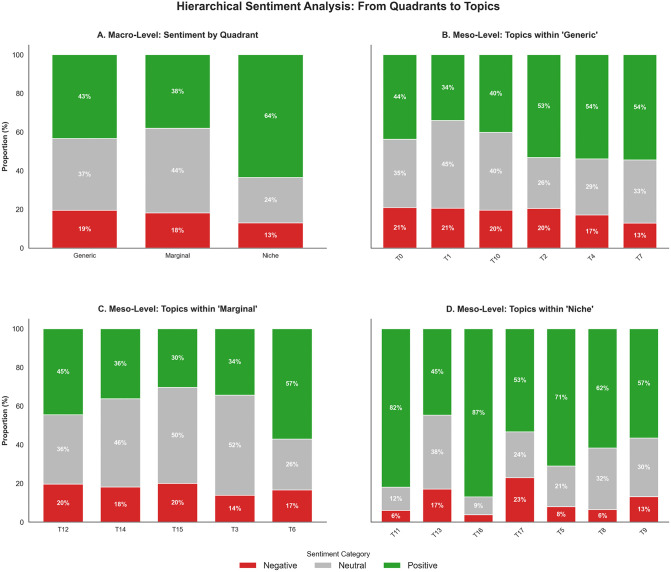
Quadrants-based distribution across topics.

Moving from the corpus-wide baseline to the quadrant level, Fig 6 reveals distinct patterns of emotional stratification. At the macro level (Fig. 6), niche topics are characterised by notably high positive sentiment (64%) and the lowest negative sentiment (13%) among all quadrants. In contrast, generic and marginal topics display more balanced sentiment distributions, with higher proportions of both neutral and negative responses, visually reflecting clear differences in emotional engagement across quadrants.

At the meso level (Fig 6), negative sentiment proportions within the generic and marginal quadrants remain highly stable, ranging from 13% to 21%. The niche quadrant (Fig 6), however, displays significant internal variation: positive sentiment for Topic 16 peaks at 87%, while negative sentiment for Topic 17 reaches 23%. This pattern indicates that, unlike the stable negative consensus observed in broadly oriented topics, reception of niche topics is markedly polarized between concentrated praise and concentrated criticism.

Chi-Square tests were conducted to verify that these patterns reflect genuine associations between reader sentiment and topic type rather than random variation. Macro-level comparisons (Table 5) confirm a highly significant difference in overall sentiment distribution across quadrants (χ² = 93.5, p < 0.001), statistically validating the emotional stratification observed in Fig 6 A. Meso-level tests (Table 6) reveal contrasting internal dynamics: although positive sentiment varies significantly within all three quadrants, negative sentiment functions as a critical differentiator — it is statistically homogeneous within the generic (p = 0.193) and marginal (p = 0.184) quadrants, yet exhibits significant heterogeneity within the niche quadrant (p = 0.003).

**Table 5:** Chi2 test results across quadrants.

Test Scope	Sentiment Polarity	χ2	p-value
**All Quadrants**	Overall Heterogeneity	93.5	< 0.001
	Positive	85.63	< 0.001
	Neutral	60.23	< 0.001
	Negative	9.55	0.008

While Fig 6 offers intuitive patterns of sentiment distribution, this study further employed Chi-Square tests to rigorously validate the association between reader sentiment and topic distribution, thereby eliminating the possibility of randomness. Initially, macro-level comparisons (Table 5) reveal a highly significant difference in overall sentiment distribution across quadrants, statistically confirming the emotional stratification observed in Fig 6A. Subsequently, meso-level tests (Table 6) uncover contrasting internal dynamics. Although positive sentiment varies significantly within all quadrants, negative sentiment emerges as a critical differentiator: it is statistically homogeneous within the generic and marginal quadrants, yet exhibits extreme heterogeneity and severe fluctuation exclusively within the niche quadrant.

**Table 6:** Chi2 test results across topics inside quadrants.

Quadrant	Sentiment Polarity	χ2	p-value
**Generic Topics**	Overall Internal	70.1	< 0.001
	Positive	59.22	< 0.001
	Neutral	48.69	< 0.001
	Negative	7.4	0.193
**Marginal Topics**	Overall Internal	61.18	< 0.001
	Positive	47.92	< 0.001
	Neutral	47.05	< 0.001
	Negative	6.21	0.184
**Niche Topics**	Overall Internal	44.22	< 0.001
	Positive	34.64	< 0.001
	Neutral	18.71	0.005
	Negative	19.85	0.003

To elucidate the cross-cultural reception mechanisms of Waley’s translation systematically, this study constructs a multidimensional analytical framework that integrates semantic similarity with topic prevalence and distinctiveness. The framework operates along two interconnected axes. First, hierarchical clustering — based on semantic similarity — reveals *what* readers are discussing by mapping the convergence of reader discourse at the structural level. Second, the quadrant matrix — utilizing topic prevalence and distinctiveness — demonstrates *how widely* these topics are discussed and *how specifically* they are expressed in lexical terms.

Applying this framework to the full topic set, the 18 topics are distilled into four core analytical dimensions that capture the principal mechanisms of reader reception (Table 7):

**Table 7:** Analytical dimensions and topic mapping of reader reception mechanisms.

Analytical Dimension	Included Topics
**Dimension 1: Translation Style and Reading Experience**	T11, T16, T17
**Dimension 2: Character Shaping and Transmedia Resonance**	T1, T2, T10
**Dimension 3: Translation Strategies and Philosophical Resonance**	T3, T14
**Dimension 4: Textual Reconstruction and Multimodal Experience**	T0, T4

### 4.4. Qualitative close reading of the reception mechanism

Building on the four analysis dimensions identified, this section delves into specific review texts to explore the underlying factors driving emotional response differences. This section analyzes only positive and negative comments, following a common paradigm in SA [47]. Neutral comments often contain a mix of positive and negative elements, which can obscure clear emotional trends and complicate the analysis. Focusing on the extremes provides sharper thematic contrasts between the divergent reader groups.

#### 4.4.1. Dimension 1: Translation style and reading experience.

Within Dimension 1 (Topics T11, T16, and T17), positive reception centres on the immersive reading experience that Waley’s translation creates. Topics T16 and T11 record particularly high positive evaluation rates of 87% and 82%, respectively. This immersive quality rests on three interconnected mechanisms from Narrative Transportation Theory: emotional arousal, reduction of cross-cultural psychological distance, and effective management of cognitive load.

First, the translation draws readers into the narrative through strong emotional engagement. Narrative transportation — the psychological process by which readers become absorbed in a story world and temporarily set aside real-world concerns — depends on the integration of attention, imagery, and feeling [48]. Waley’s rendering preserves the text’s distinctive humour, which the early critic Turner (1942) compared favourably to *Don Quixote*, praising its “all-prevailing intellectual vigour” as uniquely capable of drawing Western readers into an unfamiliar cultural landscape. This humour acts as the primary mechanism through which readers lower their cultural defences and enter the narrative world. As the reader Maxwell Lyons (2022-12-01, Topic 11) put it, the text is “so different from anything I have read before, enthralling and hilarious and wonderful.” Such engagement triggers event-congruent emotions (ECEs) — affective responses aligned with the narrative’s emotional arc [49] — which occupy cognitive resources and reduce readers’ inclination toward critical scrutiny [50,51].

Second, narrative transportation dissolves the psychological distance between reader and text. Green and Appel [52] note that once readers are transported into a narrative world, real-world markers — including the sense that a text is historically remote — are automatically suspended. This explains why Western readers engage with a centuries-old Chinese classic as though it were contemporary. The reader Abby (2019, Topic 16) observed: “Some parts are incredibly funny, and even though it was written over 500 years ago, it reads as very fresh and modern.” As Ooms et al. [53] argue, perceived psychological similarity between reader and character is more important for identification than objective demographic matching: as long as readers share emotional or value-based resonance with a character, the sense of distance rapidly collapses.

Third, the translation sustains immersion by reducing cognitive load. Maintaining narrative engagement requires continuous cognitive effort; excessive demands on working memory disrupt transportation and cause disengagement [54]. Waley’s short-chapter structure directly addresses this risk. As the reader John Stults (2022-02-13, Topic 17) noted: “The chapters are quite short, so it’s perfect if you only have a few minutes to read or are reading right before bed.” This structural lightness, combined with high narrative coherence, produces what Schwarz [55] describes as processing fluency — a condition under which readers naturally experience positive affect and attribute greater aesthetic value to what they read.

In contrast, negative feedback in Topic T17 (23% negative rate) centres on reading motivation rather than textual quality, reflecting a shift in the social context of reading. When Waley’s translation enters the university classroom, the logic governing reading changes fundamentally. Bourdieu & Wacquant [56] describe a social field as a network of objective relations defined by its own rules; Thomson [57] characterizes it as a space in which agents occupy positions and follow field-specific conventions. However, a fundamental displacement of the field occurs when the translation of *Journey to the West* enters the university classroom. As observed by Louise Webber [58], students within the higher education field must internalize a specific set of “rules of the game”—how to act as a university student, meet established academic expectations, and integrate into the academic community. Tilbrook and Shifrer [59] note that cultural capital is intrinsically field-specific. In the original “field of cultural production” where literary works reside, a logic of the “economic world reversed” dominates, emphasizing artistic autonomy and pure aesthetic intention in this space [60], readers’ engagement typically stems from aesthetic dispositions driven by their embodied cultural capital [61]; in this institutionalized context, reading behavior shifts from “willing engagement” to the instrumental pursuit of institutionalized cultural capital, such as academic credits and degrees — grades, credits, and credentials [62]. Walsh and Antoniak [29] argue that universities convert literary works into required commodities through syllabi, conferring legitimacy while simultaneously commodifying the reading experience. As the reader Jenna Laiche (2016-02-23, Topic 17) stated: “I was forced to read this book for a college course...” This resistance exemplifies what Cremin and Scholes [63] term the displacement of “volitional reading” by a “performative agenda” — an accountability-driven logic that prioritises measurable outcomes over genuine engagement. From the perspective of Self-Determination Theory, external assessment pressures suppress readers’ needs for autonomy and competence, converting reading into mechanical compliance [64]. Green and Appel [52] further note that analytical reading instructions — standard in academic settings — are inherently antagonistic to narrative transportation, prompting a critical stance that blocks immersion. Reader Ginger (2014-10-14, Topic 17) captured this clearly: “This is not a quick read, and to keep up with the class, it was hard to get through the 300 + pages in time.” As Cremin and Scholes [63] conclude, instrumentalising reading to serve academic outcomes “marginalises the subject reader and ignores the affective and social dimensions of engagement,” producing alienation rather than appreciation.

#### 4.4.2. Dimension 2: Character shaping and transmedia resonance.

Within Dimension 2 (Topics T1, T2, and T10), reception is broadly positive, with approval rates of 44%, 34%, and 53%, respectively. Positive responses focus primarily on reader identification with the Monkey King and the text’s visually dynamic action sequences. Two mechanisms drive this cross-cultural engagement: schema refamiliarisation with an unfamiliar character type, and transmedia resonance with contemporary visual culture.

Positive evaluations concentrate overwhelmingly on Monkey himself. Readers favour descriptors such as “superhero,” “mischievous,” and “intelligent,” reflecting deep appreciation for his rule-breaking energy and moral complexity. This response is shaped in part by Waley’s structural choices: as Hao [6] notes, Waley’s abridgement strips away much of the Buddhist philosophy associated with Tripitaka, shifting narrative weight toward Monkey and transforming a multi-character religious allegory into a personal coming-of-age story better suited to Western reading conventions. Monkey’s appeal is further grounded in his resemblance to the Western trickster archetype — a figure who, as Hodges et al. [65] observe, possesses boundless transformative ability and a tendency to turn rules and order into productive chaos. Readers process this unfamiliar character through “schema refamiliarization” [66]: the celestial hierarchy Monkey disrupts in “Havoc in Heaven,” conventionally read in Chinese tradition as a challenge to cosmic order, is reinterpreted by Western readers — shaped by values of individualism — as an oppressive institution suppressing personal freedom. Monkey’s rebellion thus activates a familiar heroic narrative of resisting tyranny and pursuing liberation. As Liang et al. [67] argue through a Jungian lens, Sun Wukong embodies the “child archetype” — impulsive, authentic, and morally ambiguous — whose vitality generates strong vicarious engagement. Characters with moral complexity and behavioural flaws subvert expectations of virtuous protagonists, producing the cognitive conflict and moral ambivalence that deepen narrative engagement [68].

Beyond the text itself, readers’ aesthetic responses are shaped by their exposure to contemporary visual media. As Jauss [69] argues, reception is governed by a “horizon of expectations” — a pre-structured framework formed by prior aesthetic and cultural experience. In the contemporary transmedia context, this framework manifests as a “narrow literary horizon of expectation formed based on past aesthetic experiences” [70] which establishes “a relatively certain boundary that defines the possible limits of understanding a work” [71]. For Western readers whose perceptual habits have been shaped by anime and action-oriented media, this horizon is pre-loaded with high-intensity imagery and kinetic pacing. The reader Bradley (2025-09-15, Topic 1) noted: “The fight scenes were incredible and remind me of my forays into anime,” and George Jones (2025-06-07, Topic 10) praised the novel’s “non-stop, epic, Dragon Ball-esque action.” Drawing on Hutcheon’s distinction between “showing” and “telling” modes [72]; the “showing” mode offers immediate, multi-sensory immersion through direct perception of a fictional world, whereas the “telling” mode relies on words to guide the imagination, requiring a conceptual or imaginative leap to achieve visualization. Confronted with a classical text, contemporary audiences leverage their reservoir of visual experiences to successfully execute this “imaginative leap,” vividly translating textual “telling” into high-intensity visual “showing” within their minds — achieving what Xu et al. [70] describe as active transmedia resonance, in which readers mobilize familiar action logics to bridge cultural and temporal distance.

However, not all readers achieve this positive resonance. A stable minority (21–22%) across all three topics report persistent negative reception, reflecting resistance rooted in the clash between cultural expectations and the actual affordances of the text. Cave [73], building on Gibson’s concept of “affordance—possibilities for action” [74], argues that literary texts offer “cognitive affordances” shaped by readers’ cultural habits. Western readers approach Chinese classical fiction with what Bruner calls a “canonical script” [75] — an implicit expectation of how a sacred or pious narrative should unfold and how characters should behave. As Lefevere [76] notes, all translation reflects a specific ideology and poetics operating within a given cultural context; within this context, English readers naturally construct expectations shaped by Western religious and heroic conventions. These expectations are consistently frustrated. At the level of narrative tone, reader Jonahs Ruiz (2025-02-18, Topic 2) admitted: “I went into this story with the expectation of it being a religious text and hooooo boy was I wrong.” As Hodges et al. [65] note, the novel repeatedly deconstructs the sacred — most pointedly by depicting the holy site of Vulture Peak as a bureaucracy openly demanding bribes — producing cognitive dissonance for readers who expect reverence. At the level of character, the text resists the Western hero’s journey framework outlined by Campbell [77], which requires trials, symbolic death, and genuine internal transformation. Concurrently, Western narratives accustom readers to heroes who, despite being initially “egocentric, arrogant, and disconnected from the world” [78], ultimately achieve profound internal growth through life-and-death trials. Yet, the atypical characterization in Journey to the West directly invalidates this identification mechanism. Reader Alex (2025-05-27, Topic 10) expressed this directly: “honestly, our ‘hero’ is honestly just a wildly unlikeable jerk while also being all but invincible.” Monkey’s near-invincibility renders the “Ordeal” stage effectively meaningless, and without the expected arc of psychological growth, readers lose the identification mechanisms on which narrative engagement typically depends. Together, the text’s secular mockery of religious convention and its “anti-heroic” protagonist mount a sustained challenge to readers’ canonical scripts, leaving them without familiar anchors of identification and generating the persistent negative sentiment observed across these topics.

#### 4.4.3. Dimension 3: Translation strategies and philosophical resonance.

Within Dimension 3, positive reception reveals convergent focal points with divergent value orientations. Topics 3 and 14 both attract positive responses (34% and 36%, respectively), but readers in each group engage with the text’s cultural and philosophical content in markedly different ways.

Readers in Topic 3 recognize the text’s cultural and religious dimensions but engage with them lightly, treating Buddhist elements as one component of a rich narrative blend rather than as its philosophical core. As the reader Noah (2025-06-08, Topic 3) described it, the integration of “adventure, humor, Buddhist allegory and fantastic creatures” is what makes it an “epic read.” This pattern reflects what Damrosch [1] identifies as characteristic of world literature reception: readers are aware of the original cultural context but tend to hold it lightly, engaging with the work primarily as narrative. Waley facilitated this by deliberately reducing the text’s cultural load: as he acknowledged in his preface, the source novel combines “folklore, allegory, religion, history, anti-bureaucratic satire, and pure poetry” [79]; to maximize accessibility, he translated selected prose “almost in full” while omitting most of the verses, which he judged would “go very badly into English” and diminish readability [9]. By removing culturally dense verse while preserving narrative momentum, Waley guided Western readers toward the text without overburdening them with its full complexity.

Readers in Topic 14 engage more deeply, reading the text as a vehicle for philosophical reflection and broader life metaphor. Reader A. Czepyha (2023-12-31, Topic 14) described it as “a wonderful metaphor for one’s Buddhist journey through life to enlightenment,” noting that “faith, practice, and study are main features of this novel as Tripitaka’s lovable disciples learn to improve and elevate their mind/character.” As Boxer et al. [80] suggest, this response often stems from readers’ identification with the Buddhist concept of Dependent Arising — the recognition that individual growth is embedded in collective relationships. The same reader made this connection explicit: “Personal strength (limitations) is mentioned quite a few times... reminding the reader that the journey of life is most successful when you have the company and assistance of others.” This deeper engagement reflects what Damrosch calls the “double refraction” [1] of world literature: the work retains characteristics of its source culture while being reshaped by the values and needs of the receiving culture. Readers use the Eastern mythological framework as a lens through which to reflect on their own experiences of growth and self-development. This transcultural projection is grounded in what Liang et al. [67] identify as the text’s “psychological isomorphism”: Sun Wukong’s early chaos represents the narrowness of the ego, while his subsequent journey of self-restraint enacts a process of psychological integration corresponding to Jung’s concept of individuation toward the “Self” or the “synthesis of the self” [18]. Ultimately, the text does not suffer cultural attrition by detaching from its native context. Instead, it embodies the modern idea of *littérature*—aesthetically pleasing texts that, by dint of their aesthetic quality, induce profound affective and ethical responses [81]. Western readers can adopt this foreign mythology as a personal metaphor precisely because it traces a universal psychological trajectory — a dynamic that aligns with Damrosch’s argument that world literature “gains in translation” [1], acquiring broader resonance as it moves into new cultural contexts.

Negative evaluations in both topics (14% and 18% for Topics 3 and 14, respectively; p = 0.184) converge on a single point: insufficient paratextual support for navigating the text’s cultural density. Reader Blake Shields (2025-01-24, Topic 3) noted: “I only wish some of the religious concepts and terminologies were explained better,” while reader Thu Nga Le (2020-08-01, Topic 14) remarked that “these names sound blunt in English translation,” adding that “because I am not Chinese, I spent a lot of time reading background explanations.” Genette [82] defines paratext as a “threshold” mediating between text and reader; Freeth [83] extends this to argue that paratextual elements — prefaces, footnotes, and glossaries — function as a “display window” that makes the translator’s cultural labour visible. The frustration readers experience here stems directly from Waley’s deliberate minimisation of such mediation. Hao [6] and Wang et al. [8] explain that Waley’s primary aim was to provide wartime readers with accessible, escapist entertainment. Propelled by this subjective motivation, Waley executed a series of radical, popularizing interventions both inside and outside the text. As Wang and Humblé [84] observe, “Arthur Waley (1889–1966) adopted a secularized approach.  ... He filtered out most of the religious significance, preserving the hilarious aspects.” Accordingly, he defined the novel in his introduction as “humorous folklore” and avoided inserting cultural explanations into the main text. As Anthony Yu [85] observed, this led to “radical revisions of language and vast omissions of terminology, episodes, and recurrent verse” — a pattern confirmed quantitatively by Tao et al. [86], who found that Waley retained only 11 annotations across the entire volume. By minimising paratextual mediation, Waley lowered the initial threshold of access but left contemporary readers inadequately equipped to engage with the text’s deeper cultural layers.

#### 4.4.4. Dimension 4: Text reconstruction and multimodal reception.

Within Dimension 4 (Topics 0 and 4), positive reception reflects “convergence in affective response alongside divergence in evaluative focus.” Both topics express consistent approval of the translation’s entertainment value and readability (44% and 54% positive, respectively), but with different emphases: Topic 0 focuses on textual decisions — abridgement mechanics, character naming, and the trade-off between accessibility and scholarly rigour — while Topic 4 centres on affective and multimodal experience.

Readers in both topics affirm Waley’s success in producing an accessible and entertaining text. Regarding readability, approximately 63 comments in Topic 0 employ terms such as “fun,” “accessible,” and “engaging.” Reader Daz (2025-07-25, Topic 0) praised it as an excellent “flowing story” whose pacing significantly lowers the barrier to entry for Western readers approaching a Chinese classic. Regarding entertainment value, the comedic spirit of the original achieves wide cross-cultural resonance: reader Sean Ferrell (2019-08-21, Topic 4) described the translation as “hilarious and full of fantasy, which is exceedingly rare in epic works of this kind.” Together, narrative momentum and comedic tone constitute the core of Waley’s cross-cultural appeal.

Regarding the trade-off between readability and scholarly accuracy, readers generally affirm Waley’s pragmatic positioning. Reader Patrick (2017-04-12, Topic 0) acknowledged that while Waley’s version may not be as “magisterial” as an unabridged translation, it successfully avoids the “stiffer” academic tone of the latter; he regarded it as “abridged but still meaningful” and “just right for an introduction.” On character naming, Ping and Wang [11] note that the source text contains extensive technical terminology for demons, martial techniques, and religious and alchemical concepts; Waley responded with an explicit domesticating strategy, rendering these into English equivalents familiar to Western readers. Reader Josh (2025-06-08, Topic 0) praised Waley’s “incredible decisions for personal names,” citing examples such as “Cloud Boy and Mist Lad” and “Load of Gold and War Boy.” By stripping away metaphysical connotations and foregrounding the most intuitive surface qualities of each character, Waley achieved what Lovell [87] describes as names that have “become so widely known” and retain “immense charm and vitality.”

Readers in Topic 4 emphasize the affective and sensory dimensions of their engagement. As Liu [88] argues, the cross-cultural reception of world literature increasingly encompasses not only interlingual translation but also cross-semiotic and cross-modal reconstruction; reader engagement with foreign literature involves auditory, visual, and other sensory responses that produce affective reactions such as pleasure and nostalgia. Reader Xenia (2025-12-09, Topic 4) described the text as “a typical adventure novel... but also so incredibly delightful, nostalgic & whimsical! reminded me a lot of my childhood.” As Kneuer [89] notes, nostalgia is typically triggered through multiple points of connection built on prior experience; Ingram and Luckett [90] add that such connections often take the form of parasocial relationships in which character identification is a defining feature. The cognitive accessibility inherent in books triggers these parasocial relationships through reading pleasure and a sense of presence [91]. Shedlosky-Shoemaker et al. [92] further observe that narrative transportation positively predicts cognitive overlap between reader and character, narrowing perceived psychological distance. By discarding lengthy verse and esoteric religious content in favour of brisk narrative, Waley facilitated readers’ psychological projection onto the characters, awakening latent desires for adventure and rebellion.

Negative feedback in Topic 0 (21%) centres on perceived plot repetition, reflecting a structural mismatch between Eastern and Western narrative conventions. As Salesses [93] argues, narrative form is culturally determined: the shape of a story reflects the expectations of its cultural context. Western readers are typically conditioned by Freytag’s Pyramid — a linear structure built around a single conflict progressing through rising action, climax, and resolution. The Ming dynasty hundred-chapter *Journey to the West*, which served as the source text for Waley’s translation [94], by contrast, operates on what might be described as a “fishbone diagram” structure [95]: the scripture-seeking mission forms a central narrative spine, while individual demon-subduing episodes unfold as modular, self-contained units. As Ryder [96] notes, the fundamental structural principle of the Ming novel is “digression” — a “string of xiaoshuo (小说)” in which episodes function as “links in a chain.” This patterned repetition was originally a feature of oral storytelling, designed to generate rhythmic tension and release for a listening audience. Although Waley condensed the text into thirty chapters, this shortened the chain without changing its interlinked nature. When readers expect linear narrative progression but encounter the episodic *zhanghui* (章回) structure instead, the repetition mechanism — effective in its original oral context — becomes a source of cognitive friction that impedes aesthetic engagement.

Negative feedback in Topic 4 (17%) focuses on the audiobook narration. Reader Tien (2024-12-07, Topic 4) commented: “The very British narrator really threw me off.” Pérez-González [97] notes that when processing audiovisual content, listeners first process the form of words before mapping meaning onto them; in an audiobook context, the narrator’s accent and intonation constitute the primary auditory form through which meaning is received. This form activates listeners’ prior cultural associations: the British accent immediately triggers expectations of Victorian literature or Shakespearean drama [98], creating a fundamental mismatch with the Chinese mythological content of the text. Successful audiovisual adaptations of Chinese mythology — such as the English trailer for *Monkey King: Hero Is Back*, which cast Jackie Chan as voice actor — leverage culturally congruent prior associations to facilitate coherent cognitive mapping [99]. In Waley’s audiobook, however, the publisher’s decision to employ a standard British narrator — intended to lower the barrier of acceptance through target-culture orientation — overlooked the cultural identity embedded in the voice itself. As Kosch et al. [100] observe, a narrator’s “material voice” makes its inherent cultural associations highly salient, suppressing listeners’ neutral internal voice and constraining their imaginative space. The result is a fundamental incompatibility between auditory form and textual content that disrupts cognitive mapping and collapses cross-cultural immersion.

## 5. Conclusion

This study applied a joint BERTopic–Twitter-RoBERTa computational framework to 796 English-language reviews of Arthur Waley’s *Monkey: Journey to the West*, systematically collected from Goodreads and Amazon. Topic modeling identified 18 distinct topics, subsequently consolidated into four analytical dimensions: Translation Style and Reading Experience; Character Shaping and Transmedia Resonance; Translation Strategies and Philosophical Resonance; and Textual Reconstruction and Multimodal Reception. Across all four dimensions, positive sentiment was dominant (approximately 60%), confirming that Waley’s accessible, colloquial prose and vivid characterization of the Monkey King generated sustained cross-cultural appeal.

Analysis of the four dimensions revealed that positive reception was driven by four factors: Waley’s colloquial prose style, which lowered the cognitive barrier to engagement; Sun Wukong’s trickster-hero appeal that resonated with Western heroic expectations; Buddhist allegory, which generated cross-cultural philosophical resonance; and the translation’s humor and nostalgic entertainment value. Negative reception stemmed from five aspects: intrinsic motivation and aesthetic immersion suppressed by institutional reading contexts, Monkey’s invincible and non-developing characterization that violated Western hero’s journey expectations; Waley’s minimal paratextual strategy, which made it difficult for readers unable to decode cultural and religious content; the *zhanghui* episodic structure, which clashed with Western linear narrative expectations; and the audiobook’s British-accented narration that disrupted cross-cultural cognitive mapping. Together, these findings demonstrate that cross-cultural literary reception is shaped not by translation quality alone, but by the interplay between translation strategy, reader schema, and multimodal reception.

At the practical level, this evidence suggests that effective dissemination of classical Chinese literature in high cultural-distance markets requires a dual-channel approach: maximising textual accessibility and entertainment value while simultaneously deploying paratextual resources to contextualize deeper cultural and philosophical content for readers who seek it. The cross-cultural reach of Waley’s translation further supports Damrosch’s model of world literature as “elliptical refraction”: Waley’s mediation did not eliminate cultural specificity but reframed it, enabling transcultural resonance without requiring readers to possess prior knowledge of the source tradition. Overseas literary communication is thus not merely a matter of linguistic conversion, but of cultural meeting and renewal.

Several limitations of this study warrant acknowledgement. The corpus is restricted to two English-language platforms, so the findings represent one specific cultural-linguistic reception context and cannot be directly generalized to other linguistic communities. The Twitter-RoBERTa sentiment model, though validated and found to perform reliably on this corpus, classifies reviews into broad positive, negative, and neutral categories; it cannot resolve finer-grained distinctions within sentiment — such as distinguishing admiration from nostalgia, or frustration from disappointment — that would enrich the interpretive depth of the findings. While the PDR–PRM refinement improved BERTopic’s topic coherence, thematic boundaries in literary discourse are inherently fuzzy and automated methods cannot fully resolve cross-topic overlaps. Finally, reviewer demographics are unavailable: the sample represents self-selected, digitally engaged audiences and may not reflect the full spectrum of reader reception. Future research could address these constraints by incorporating multilingual review corpora, applying domain-adapted sentiment models, and integrating demographic metadata, to produce a more comprehensive cross-cultural account of classical Chinese literary circulation.
